# A Nano-Pharmaceutical Formula of Quercetin Protects from Cardiovascular Complications Associated with Metabolic Syndrome

**DOI:** 10.3389/fphar.2021.696981

**Published:** 2021-08-11

**Authors:** Osama A. A. Ahmed, Noura A. Hassan, Ahmad S. Azhar, Mahmoud M. El-Mas, Hany M. El-Bassossy

**Affiliations:** ^1^ Department of Pharmaceutics, Faculty of Pharmacy, King Abdulaziz University, Jeddah, Saudi Arabia; ^2^ Mohamed Saeed Tamer Chair for Pharmaceutical Industries, King Abdulaziz University, Jeddah, Saudi Arabia; ^3^ Department of Pharmacology and Toxicology, Faculty of Pharmacy, Zagazig University, Zagazig, Egypt; ^4^ Pediatric Cardiac Center of Excellence, Faculty of Medicine, King Abdulaziz University, Jeddah, Saudi Arabia; ^5^ Department of Pharmacology and Toxicology, Faculty of Pharmacy, Alexandria University, Alexandria, Egypt; ^6^ Department of Pharmacology and Toxicology, Faculty of Medicine, Kuwait University, Kuwait city, Kuwait

**Keywords:** quercetin, cardiovascular complications, metabolic syndrome, SNEDDS, nano-formulation

## Abstract

Metabolic syndrome (MetS) is closely associated with the development of cardiovascular diseases. We recently developed a nano-preparation of the flavonoid quercetin (QU) in a self-nanoemulsifying drug delivery system (SNEDDS). The latter comprised a mixture composed of pumpkin seed oil, D-α-Tocopherol polyethylene glycol 1,000 succinate and polyethylene glycol. The QU SNEDDS preparations exhibited a considerably higher bioavailability compared with the standard quercetin suspension. Here, we investigated whether the quercetin loaded SNEDDS could offer better protection compared with the standard formulation against cardiovascular complications of MetS in rats. MetS was induced by high fructose, high salt and high fat diet for 12 weeks while the nano-preparation or the standard suspension of quercetin was orally administered for the last 6 weeks. Compared to little effect for the standard quercetin suspension (MQ), the treatment of MetS rats with the quercetin loaded SNEDDS (MNQ) virtually abolished the depressant effect of MetS on contractility index (control, 114 ± 4; MetS, 92 ± 3; MQ, 100 ± 2; MNQ, 114 ± 6 1/s) and rate of rise in left ventricular pressure (dP/dtmax) (control, 8,171 ± 274; MetS, 6,664 ± 135; MQ, 6,776 ± 108; MNQ, 7,498 ± 303 mmHg/s). Likewise, the prolongation by MetS of electrocardiographic markers of arrhythmogenesis (QTc, JT, and Tpeak-to-Tend intervals) and concomitant rises in dicrotic notch pressure were preferentially reversed by quercetin nano-preparation. On the other hand, the rises in the isovolumic relaxation constant (*Tau*, denotes diastolic dysfunction), blood pressure, pulse pressure, and difference between systolic and dicrotic pressure (SDP difference) were equally improved by the two preparations of quercetin. Additionally, no differences were noted in the ability of the two quercetin preparations in abrogating the elevated oxidative (MDA) and inflammatory (TNFα) markers in cardiac tissues of MetS rats. Histopathological, microscopical signs of necrosis, inflammatory cell infiltration, and vascular congestion in MetS hearts were more markedly inhibited by the nano-preparation, compared with the standard preparation of quercetin. In conclusion, the quercetin loaded SNEDDS is evidently more advantageous than the standard preparation of the drug in alleviating functional and histopathological manifestations of cardiac damage incited by MetS.

## Introduction

Metabolic syndrome (MetS), which is also known as insulin resistance syndrome or syndrome X, is denoted by a group of conditions, namely central obesity, dyslipidemia, hypertension, and hyperglycemia (Alberti et al., 2005). MetS is generally considered injurious due to its linkage with the cardiovascular diseases (CVD) and type 2 diabetes mellitus. Further, a key signal of the related cardiovascular complications is vascular damage and irregular vascular response to vasoconstrictors (facilitation) as well as vasodilators (inhibition) (Bahia et al., 2006). Hypertension, arterial wall stiffness, dyslipidemia, and atherosclerosis are some prominent features of MetS that underlie the provoked vascular damage and dysfunction (Oliver and Webb, 2003; Olijhoek et al., 2004).

The management of MetS relies traditionally on the use of therapies that improve major complications of the disease such as hyperglycemia, hyperlipidemia, and hypertension. Treatment with common medications exerts unpleasant adverse effects such as myalgia, gastritis, hepatitis, hypoglycemia and hypotension in the early stage of treatment (Kaur, 2014). This high level of adverse effects leads to a weak tolerance of the patient especially in the long-term application of the medications (Taghipour et al., 2019). So there is an increasing exploration of using natural products, especially plant polyphenols, along with drugs in order to better regulate the disease (Visioli, 2011). Nanosized drug carriers, consisting of phytochemicals endowed with developed pharmacodynamics and pharmacokinetic characteristics, are a novel therapeutic approach (Taghipour et al., 2019).

Quercetin is a compound present in fruits such as apples and vegetables such as peppers and onions. Quercetin’s multiple health benefits have been widely reported for their anti-inflammation and antioxidant properties as well as increased endothelium-dependent vasodilation (Murakami et al., 2008; Jagtap et al., 2009; Shen et al., 2012). However, the application of quercetin in pharmaceutical field is limited due to its poor solubility, low bioavailability, poor permeability, and instability (Ahmed et al., 2020). Therefore, it is highly necessary to develop new dosage forms of quercetin with increased solubility and improved bioavailability (Cai et al., 2013). Self-nanoemulsifying drug delivery systems (SNEDDS) have drawn attention for their ability to improve the bioavailability and hence efficacy of orally administered substances like quercetin. SNEDDS systems consist of oil, surfactant, and co-surfactant beside the active ingredient (Yang et al., 2018).

We have previously shown that quercetin improves the compromised vascular reactivity caused by MetS or advanced glycation end-products *in vitro* (Ahmed et al., 2020). However, the *in vivo* effect of quercetin administration on different hemodynamic and electrocardiographic parameters was not evaluated. Furthermore, our team has recently developed a TPGS and PSO Based SNEDDS Formulation of quercetin with improved bioavailability and a globule size of 320 nm and zeta potential of −28.6 mV (Ahmed et al., 2021). So as a follow-up to our previous reports, the current study tested the hypothesis that the improved bioavailability of the developed SNEDDS preparation of quercetin would prompt better control of cardiovascular complications of MetS. Accordingly, studies were undertaken to assess the possible counterbalancing effects of the SNEDDS pharmaceutical formula of quercetin as compared to the standard formula on hemodynamic, electrocardiographic, left ventricular dynamic, and histopathological derangements in the high fructose/salt rat model of MetS. Further, the research was extended to determine the role of inflammatory, oxidative, and adipokine pathways in the interaction.

## Methods

### Experimental Animals

For this study, male Wistar rats, 6–8 weeks old, weighing 180–200 g (Zagazig University, Zagazig, Egypt) were used. They were kept in clear polypropylene cages, four rats in each cage, and clean drinking water and rodent pellets were also provided. Under the animal housing conditions, alternating 12 h of light and dark was employed, at 22 ± 3°C temperature, 50–60% relative humidity, and sufficient ventilation. The experimental model and animal handling process were followed as per the Ethical Committee for Animal Handling guidelines, Zagazig University (ZU-IACUC/3/F/199/2019).

Animals were randomly divided into five experimental groups. Group 1: control rats (C) that received regular tap water and food pellets for 12 consecutive weeks. Group 2: MetS rats (M) that received fructose (10%) in drinking water and NaCl (3%) and high-fat diet (25%) in food pellets for 12 consecutive weeks. Group 3: rats received a single daily dose (83 μmol/kg) of the standard quercetin suspension (MQ) during the last 6 weeks of the study. Group 4: rats received a daily dose of the nano-formula vehicle (MN) in the same volumes of SNEDDS ingredients (Pumpkin seed oil, D-α-tocopheryl polyethylene glycol succinate (TPGS), and PEG 200) without quercetin during the last 6 weeks of the study. Group 5: rats received a single daily dose of the quercetin nano-formula preparation (MNQ) at a dose of 83 μmol/kg of quercetin during the last 6 weeks of the study.

### Blood Pressure Recording

For invasive recordings of blood pressure hemodynamics, the procedure described in the existing literature was followed (Azhar and El-Bassossy, 2014; El-Bassossy and Watson, 2015; El-Bassossy et al., 2017a; El-Bassossy et al., 2017b). Following the administration of anesthesia through a single intraperitoneal injection with 365 μmol/kg ketamine and 45 μmol/kg xylazine, the rats were mounted on regulated heating pads. Further, a microtip pressure transducer (SPR-320; Millar Instruments, Houston, TX, United States) was fitted into the right carotid artery. After 5 min of stabilization, the signals were noted down for 10 min. The microtip catheter was linked to a PowerLab Data Interface Module on LabChart professional software (version 8.0; AD Instruments, Bella Vista, Australia) containing a blood pressure module on a computer. The BP module was used to quantitatively study the systolic, diastolic, and dicrotic notch blood pressure.

### Cardiac Hemodynamics Recording

After 10 min of recording of blood pressure hemodynamics, the microtip catheter was advanced into the left ventricle under careful pressure control. Then the cardiac hemodynamic signals were recorded for another 10 min. The cardiac hemodynamic module was employed to quantitively determine contractility index, rate of rising in LV pressure (dP/dtmax), isovolumic relaxation constant (*Tau*), and heart rate (HR).

### Electrocardiographic Recording

The surface ECG was recorded in anaesthetized rats at the same time of cardiac hemodynamic recording by the method described previously by our group (El-Bassossy et al., 2016; El-Bassossy et al., 2017c; El-Bassossy et al., 2018a). The used system was Powerlab (ADI Instruments) connected to a PC running LabChart professional software (version 8.0) containing an ECG module, which quantitatively determines different components of the ECG wave; PR, QTc, and JT intervals.

### Biochemical and Immunologic Measurements

After cardiac hemodynamic recording, a small incision was made in the lower abdomen and 2 ml of blood were withdrawn from the inferior vena cava, allowed to coagulate before separating the serum by centrifugation (8,000 rpm, 4°C, 10 min). Aliquots of the serum were stored at −80°C for the measurement of insulin and adiponectin levels by rat specific sandwich enzyme-linked immunosorbent assay (ELISA) kits (Crystal Chem inc. Elk Grove Village, IL 60007 United States and MyBioSource, Inc. San Diego, CA, United States respectively) according to manufacturer’s guidelines.

Following blood sampling, the heart was excised and ∼50 mg from the left ventricle was snap-frozen and stored at–80°C to analyze later for tumor necrosis factor-α (TNF-α), malondialdehyde (MDA), and protein content using Mybiosource^®^ ELISA rat specific TNF-α assay kit (MyBioSource, Inc. San Diego, CA, United States) and Biodiagnostic kits for MDA and protein (Biodiagnostic^®^, Dokki, Giza, Egypt).

### Histopathologic Examination

The remainder of the heart was placed in 10% neutral buffered formalin for a duration of 2 days before being moved to 1% neutral buffered formalin for histopathology (hematoxylin and eosin stain).

### Drugs and Chemicals

The following drugs and chemicals were used in this study: Quercetin (Purity >95% by HPLC, Sigma-Aldrich, Dorset, United Kingdom), ketamine (>99%. Sigma Pharmaceutical Industries, Menoufia, Egypt), xylazine (≥99%, Sigma-Aldrich, St. Louis, MO, United States).

### Statistical Analysis

Values are expressed as mean ± standard error of the mean. Statistical comparisons were carried out using one-way ANOVA, followed by Newman-Keuls’ post hoc test using Prism 5^®^ software (Graphpad, CA, United States). Probability levels less than 0.05 were considered statistically significant.

## Results

### MetS/Quercetin Interaction on Cardiac Hemodynamics

**FIGURE 1:**
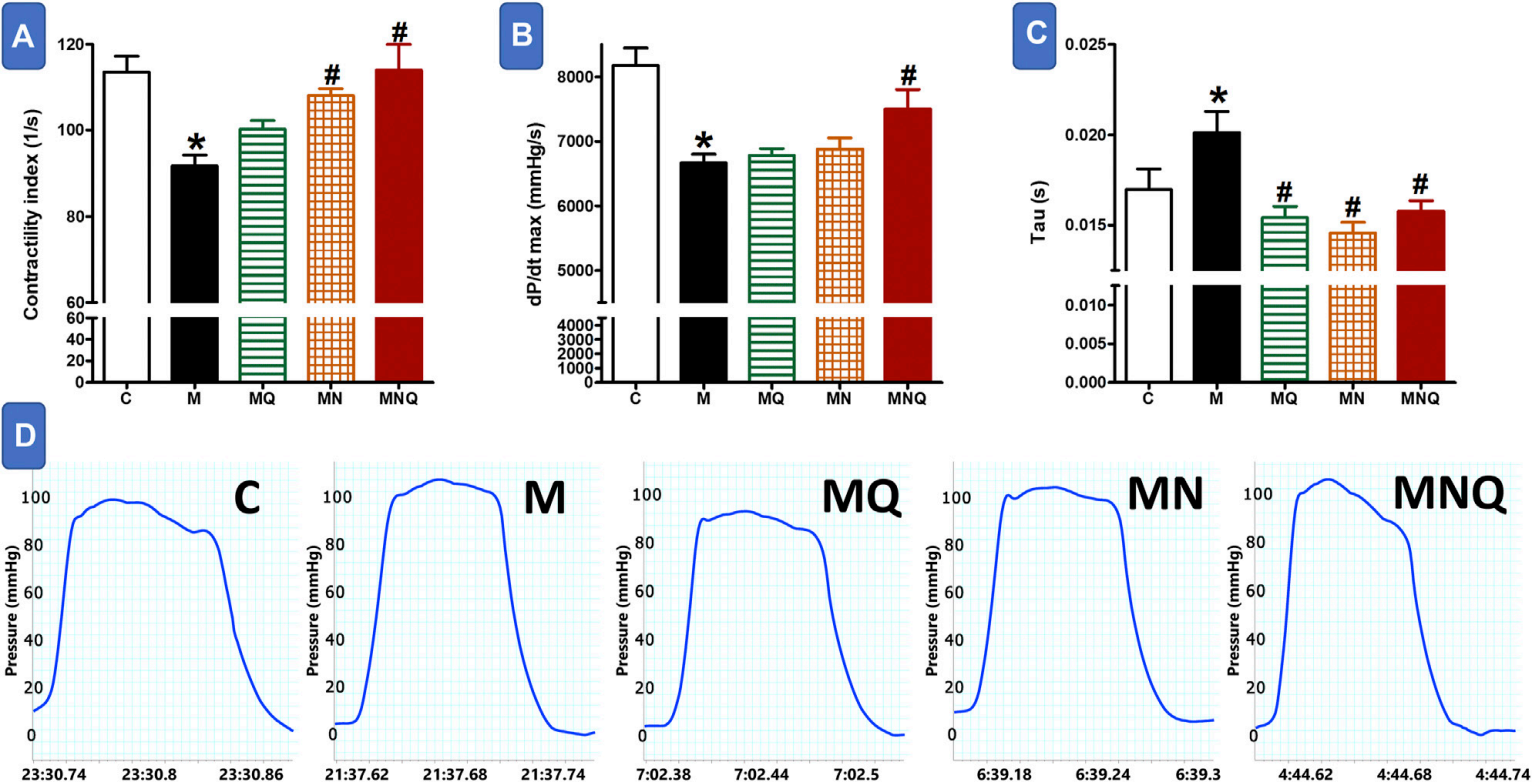
Effect of oral administration of standard quercetin suspension (MQ), plain nano-pharmaceutical formulation (MN) and the quercetin loaded nano-pharmaceutical formulation (MNQ) both at 83 μmol/kg/day on contractility index **(A)**, dp/dt max **(B)** and Tau **(C)** in metabolic syndrome (M) induced by feeding rats a high fructose (10% in drinking water), high salt (3%), high fat (25%) diet for 12 weeks. **Panel D** shows representative original recording of ventricular pressure. Results are expressed as mean ± SEM (n = 8 for all groups). *p < 0.05 when compared to the corresponding control (C) values, #*p* < 0.05 when compared to the corresponding MetS values using one-way ANOVA followed by Dunnet’s post-hoc test.

Feeding rats with a high fructose/salt/fat diet for 12 weeks resulted in marked reductions in cardiac contractility as appearing from the significant decrease in contractility index compared to control animals (*p* < 0.05, Figures 1A,D). Daily administration of standard quercetin suspension in a dose of 83 μmol/kg during the last 6 weeks did not significantly affect the reduced cardiac contractility in MetS animals. Administration of the plain nano-pharmaceutical formula significantly increased cardiac contractility in MetS but did not completely restore normal cardiac contractility (*p* < 0.05, Figures 1A,D). However, the quercetin-loaded nano-pharmaceutical formula containing the same dose of quercetin completely restored cardiac contractility to normal levels in control rats (*p* < 0.05, Figures 1A,D).

The heart of MetS animals suffered from left ventricular systolic dysfunction as reflected by a significant decrease in the rate of rising in LV pressure (dP/dtmax, *p* < 0.05, Figure 1B). The treatment with quercetin suspension or the plain nano-pharmaceutical formula failed to improve the deteriorated cardiac systolic function in MetS animals. By contrast, the quercetin-loaded nano-pharmaceutical formula almost restored systolic function to its near-control levels (*p* < 0.05, Figure 1B).

The left ventricular diastolic time constant (*Τau*) was increased in MetS animals compared to control values (*p* < 0.05, Figure 1C) indicating impairment in diastolic function. The treatment with any of the three regimens, quercetin suspension, plain nano-pharmaceutical formula, or quercetin-loaded nano-pharmaceutical formula, significantly and similarly reversed the rises in *Tau* provoked by MetS (all at *p* < 0.05, Figure 1C).

### MetS/Quercetin Electrocardiographic Interaction

**FIGURE 2:**
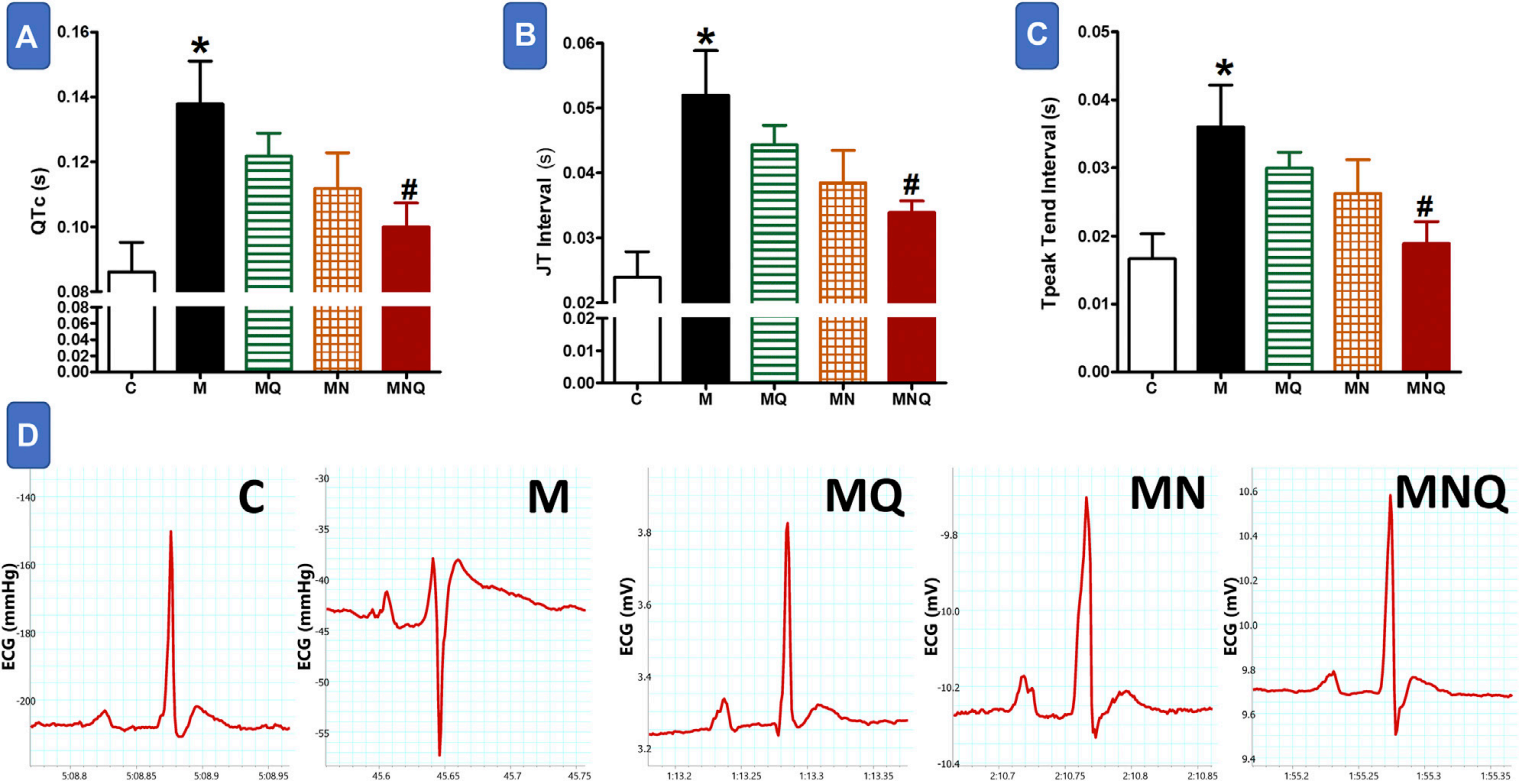
Effect of oral administration of standard quercetin suspension (MQ), plain nano-pharmaceutical formulation (MN) and the quercetin loaded nano-pharmaceutical formulation (MNQ) both at 83 μmol/kg/day on QTc **(A)**, JT interval **(B)** and PR interval **(C)** in metabolic syndrome (M) induced by feeding rats a high fructose (10% in drinking water), high salt (3%) and high fat (25%) diet for 12 weeks. **Panel D** shows representative original recording of ECG. Results are expressed as mean ± SEM (n = 8 for all groups). *p < 0.05 when compared to the corresponding control (C) values, #*p* < 0.05 when compared to the corresponding MetS values using one-way ANOVA followed by Dunnet’s post-hoc test.

Figure 2 shows that MetS induced by a high fructose/salt/fat diet resulted in delayed ventricular repolarization as reflected by a significant prolongation in QTc, JT, and T peak-Tend intervals compared to control values (*p* < 0.05). The prolonged QTc, JT, and T peak-Tend intervals were slightly, but not significantly, shortened in MetS rats treated with quercetin suspension or the plain nano-pharmaceutical formula. On the other hand, the MetS-related prolongation in the duration of these intervals was significantly reduced after treatment with the nano-pharmaceutical formula of quercetin (*p* < 0.05, Figure 2). The quercetin nano formula almost restored normal ventricular repolarization as appearing from the significant decrease in QTc, JT, and T Peak-Tend intervals (Figure 2).

### MetS/Quercetin Interaction on Blood Pressure and Heart Rate

**FIGURE 3:**
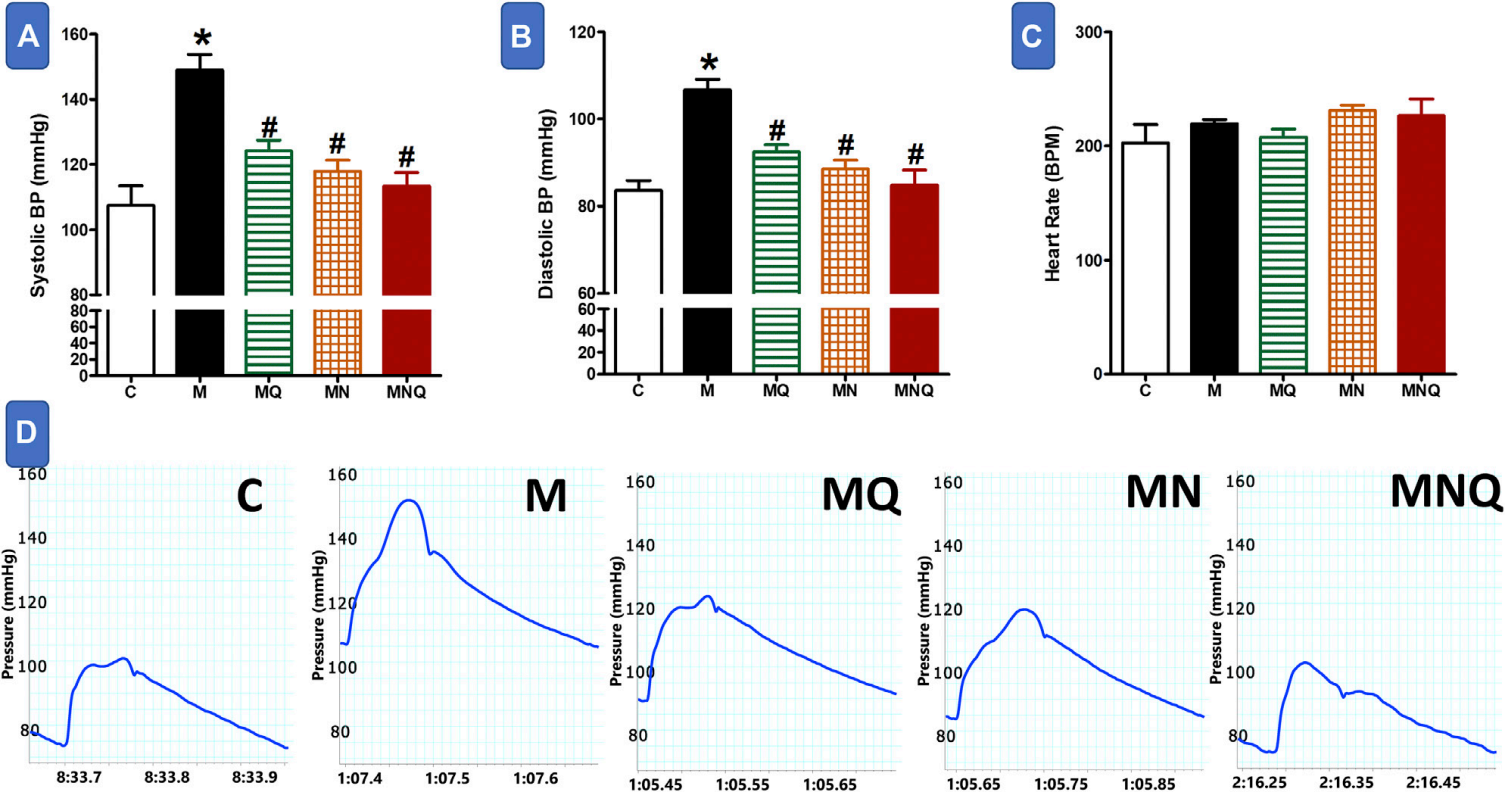
Effect of oral administration of standard quercetin suspension (MQ), plain nano-pharmaceutical formulation (MN) and the quercetin loaded nano-pharmaceutical formulation (MNQ) both at 83 μmol/kg/day on systolic BP **(A)**, diastolic BP **(B)** and heart rate **(C)** in metabolic syndrome (M) induced by feeding rats a high fructose (10% in drinking water), high salt (3%) and high fat (25%) diet for 12 weeks. **Panel D** shows representative original recording of arterial hemodynamics. Results are expressed as mean ± SEM (n = 8 for all groups). *p < 0.05 when compared to the corresponding control (C) values, #*p* < 0.05 when compared to the corresponding MetS values using one-way ANOVA followed by Dunnet’s post-hoc test.

MetS animals showed significant elevations in both systolic blood pressure (SBP) and diastolic blood pressure (DBP) compared to control animals (*p* < 0.05, Figures 3A,B,D). Quercetin administration partially reduced the elevations in SBP and DBP compared to MetS animals (*p* < 0.05). The plain nano-formula produced similar reductions in SBP and DBP (*p* < 0.05). However, the nano-pharmaceutical formula of quercetin markedly reduced the elevations in both systolic and diastolic BP nearly to control values (*p* < 0.05, Figures 3A,B,D). On the other hand, MetS animals treated with or without any of the three intervening regimens did not produce any change in heart rate compared to the control group (Figure 3C).

### MetS/Quercetin Interaction on Pulse Pressure, Dicrotic Notch Pressure, and SDP Difference

**FIGURE 4:**
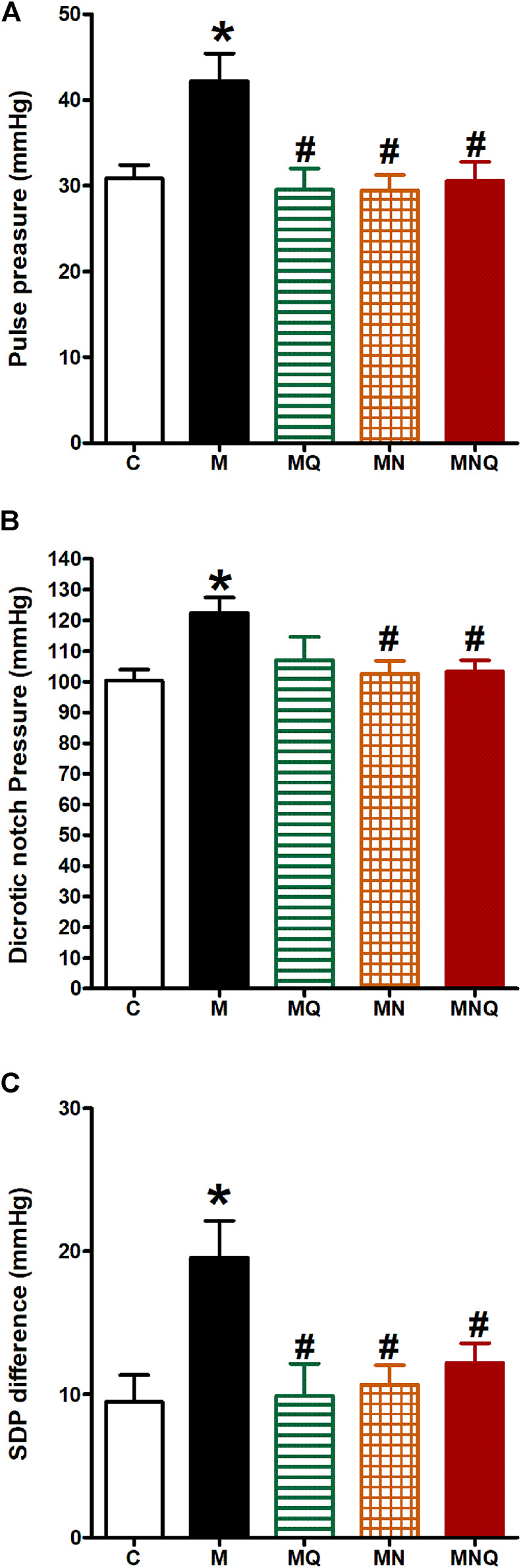
Effect of oral administration of standard quercetin suspension (MQ), plain nano-pharmaceutical formulation (MN) and the quercetin loaded nano-pharmaceutical formulation (MNQ) both at 83 μmol/kg/day on pulse pressure **(A)**, dicrotic notch pressure **(B)** and SDP difference **(C)** in metabolic syndrome (M) induced by feeding rats a high fructose (10% in drinking water), high salt (3%) and high fat (25%) diet for 12 weeks. Results are expressed as mean ± SEM (n = 8 for all groups). *p < 0.05 when compared to the corresponding control (C) values, #*p* < 0.05 when compared to the corresponding MetS values using one-way ANOVA followed by Dunnet’s post-hoc test.

MetS induction resulted in a significant increase in pulse pressure, dicrotic notch pressure, and the difference between systolic and dicrotic pressure (SDP difference, *p* < 0.05, Figure 4) compared to the control group. Quercetin administration significantly reduced pulse pressure and SDP difference, but not the dicrotic notch pressure, compared to MetS group (Figure 4). However, daily oral administration of the plain nano-pharmaceutical formulation or the nano-pharmaceutical formula of quercetin in the last 6 weeks significantly reduced pulse pressure, dicrotic notch pressure, and SDP difference compared to MetS rats (*p* < 0.05, Figure 4).

### MetS/Quercetin Oxidative Interaction

**FIGURE 5:**
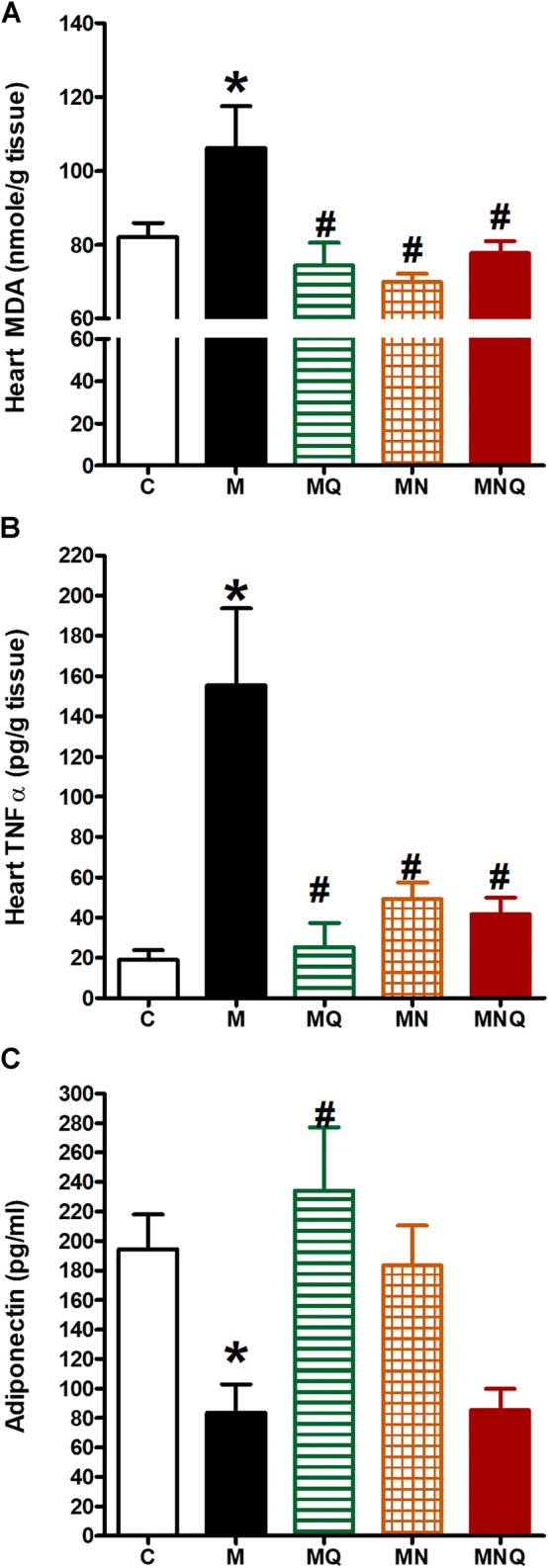
Effect of oral administration of standard quercetin suspension (MQ), plain nano-pharmaceutical formulation (MN) and the quercetin loaded nano-pharmaceutical formulation (MNQ) both at 83 μmol/kg/day on heart MDA **(A)**, heart TNF-α **(B)** and serum adiponectin **(C)** in metabolic syndrome (M) induced by feeding rats a high fructose (10% in drinking water), high salt (3%) and high fat (25%) diet for 12 weeks. Results are expressed as mean ± SEM (n = 8 for all groups). *p < 0.05 when compared to the corresponding control (C) values, #*p* < 0.05 when compared to the corresponding MetS values using one-way ANOVA followed by Dunnet’s post-hoc test.

Induction of MetS in rats caused a significant increase in heart MDA level (*p* < 0.05, Figure 5A) compared to the control group. The treatment with any of the three regimens, quercetin suspension, plain nano-pharmaceutical formula, or quercetin loaded nano-pharmaceutical formula, significantly and similarly reversed the rises in MDA level provoked by MetS (*p* < 0.05, Figure 5A).

### MetS/Quercetin Inflammatory Interaction

Induction of MetS in rats caused a significant increase in heart TNF-α level (*p* < 0.05, Figure 5B) compared to the control group which indicates that MetS is associated with low-grade inflammation. The treatment with any of the three regimens, quercetin suspension, plain nano-pharmaceutical formula, or quercetin loaded nano-pharmaceutical formula, significantly reduced the elevated level of TNF-α level caused by MetS (*p* < 0.05, Figure 5B).

Moreover, MetS induction resulted in a significant decrease in serum adiponectin level (*p* < 0.05, Figure 5C) compared to the control group. Daily oral administration of quercetin suspension at a dose of 83 μmol/kg in the last 6 weeks significantly increased serum adiponectin level compared to MetS rats (*p* < 0.05, Figure 5C). The plain nano-pharmaceutical formulation increased serum adiponectin level but did not reach the level to be significantly different from MetS. However, the nano-pharmaceutical formula of quercetin did not produce a change in serum adiponectin level compared to the MetS group (Figure 5C).

### MetS/Quercetin Interaction on Insulin and Lipid Profile

Table 1 shows that MetS rats showed significantly elevated serum insulin level compared with the control group. While, quercetin, nano-pharmaceutical formula of quercetin, and even the plain formula, all significantly inhibited the hyperinsulinemia (all at *p* < 0.05). MetS animals showed significant hypertriglyceridemia compared to control (*p* < 0.05) while only the plain formula was able to significantly inhibit this hypertriglyceridemia (*p* < 0.05). However, neither the MetS animals nor any of the treatments in the current study significantly affected the cholesterol level.

**TABLE 1:** Effect of oral administration of standard quercetin suspension (MQ), plain nano-pharmaceutical formulation (MN) and the quercetin loaded nano-pharmaceutical formulation (MNQ) both at 83 μmol/kg/day on serum insulin, triglycerides (TG) and total cholesterol (TC) levels, in metabolic syndrome (M) induced by feeding rats a high fructose (10% in drinking water), high salt (3%), high fat (25%) diet for 12 weeks.

Groups	Insulin (ng/ml)	TG (mg/dl)	TC (mg/dl)
C	1.14 ± 0.22	77.5 ± 5.31	114.1 ± 8.03
M	3.31^a^ ±0.61	162.8^a^ ±21.08	110.0 ± 6.26
MQ	1.79^b^ ± 0.47	170.8 ± 25.75	114.3 ± 7.34
MN	1.71^b^ ± 0.23	92.43^b^ ± 15.29	98.1 ± 10.45
MNQ	1.78^b^ ± 0.33	133.10 ± 13.90	130.5 ± 9.42

Results are expressed as mean ± SEM (n = 8 for all groups).

a *p* < 0.05 when compared to the corresponding control values.

b *p* < 0.05 when compared to the corresponding MetS values using one-way ANOVA followed by Dunnet’s post-hoc test.

### MetS/Quercetin Histopathologic Interaction

**FIGURE 6:**
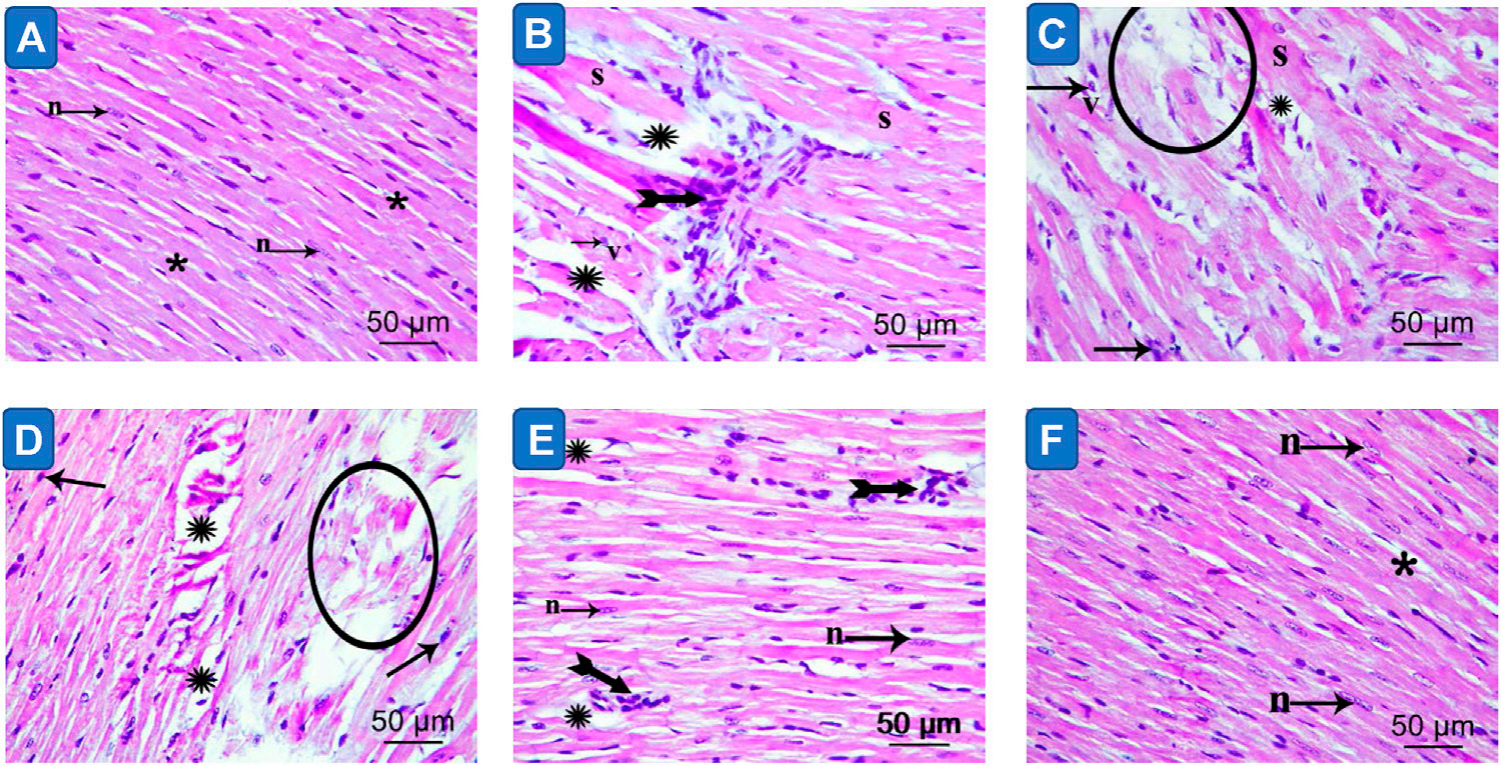
Photomicrograph of sections in the ventricular wall of control **(image A)**, Metabolic syndrome **(images B, C)**, plain formula **(image D)**, quercetin suspension **(image E)** and quercetin nano-formula **(image F)** groups. Bifid arrows represent; mononuclear cellular infiltration was evident in between the cardiac myocytes. Circle represent degenerated muscle fibers. “S” represents cardiomyocyte showing loss of striation. Small arrow represents myocytes exhibit dark stained nuclei. “v” represents monocytes with vacuolated cytoplasm (v) Scale bar = 50μm, ×400.

Light microscopy of cardiac myocytes in the control group’s ventricles presented a normal histological outcome. Myocytes were found to be branching and cylindrical in shape. An acidophilic sarcoplasm and central oval single nuclei (Figure 6A) were also seen. In MetS hearts, distinct histological changes were noted. Also, disruption and fragmentation of cardiac myocytes were apparent. Most muscle fibers showed cytoplasmic lysis, while numerous myocytes presented dark stained nuclei. The cardiac myocytes were distinguished by wide intercellular spaces. Further, mononuclear cellular infiltration was noticed intermediately among cardiac myocytes (Figures 6B,C; Table 2). The hearts presented partial restoration of the control group’s histological pattern in both plain formula (Figure 6D) as well as standard quercetin suspension (Figure 6E). Most cardiac muscle fibers were cylindrical with a central oval-shaped nuclei. However, in between cardiac myocytes, wide intercellular spaces were still noted. Some of the muscle fibers showed striations disappearing. Moreover, little mononuclear cellular infiltration could be noted in between some of the cardiac myocytes (Table 2). In the NQ group, the histological pattern was similar to that of the control group. Most cardiac muscle fibers were found to be cylindrical with a central oval-shaped nuclei. Moderately wide intercellular gaps were found between cardiac myocytes (Figure 6F; Table 2).

**TABLE 2:** Scoring of histopathological findings of hearts of control (C), metabolic syndrome (M), plain nano-pharmaceutical formulation (MN), standard quercetin suspension (MQ) and the quercetin loaded nano-pharmaceutical formulation (MNQ) groups.

Groups	Myocardial necrosis	Myocardial fibrosis	Myocarditis (inflammatory cells infiltrations)	Congested of intramuscular blood vessels
C	**--**	**--**	**--**	**--**
M	**+++**	**--**	**+++**	**++**
MQ	**++**	**--**	**++**	**++**
MN	**--**	**--**	**+**	**+**
MNQ	**--**	**--**	**--**	**--**

Semi-quantitative lesions score system was designed as follows (0 = no alterations, + = mild alterations (15–25%), ++ = moderate alterations (35–65%), +++ = severe alterations (up to 70%).

## Discussion

The current study clearly shows that the provided SNEDDS preparation significantly potentiates quercetin cardioprotective effect. While the standard quercetin suspension failed to completely protect from cardiovascular dysfunction in metabolic syndrome, the current nano-formula containing the same dose of quercetin almost completely restored normal cardiovascular function.

To study the efficacy of the current SNEDDs of quercetin on the cardiovascular function we tested its effect on left ventricular pressure measurements, ECG, and arterial hemodynamics and compared the resulted effect with that of quercetin suspension and the plain nano-formulation. Here, we evaluated the effect of the three regimens on cardiac contractility due to its importance in safety assessment studies since either an increase or a decrease may be harmful under certain clinical situations (Sarazan et al., 2012). MetS showed signs of impaired cardiac contractility as evidenced by the reduction in parameters like maximal left ventricular pressure (dP/dt max) and contractility index which can exacerbate the consequences of clinical heart failure (Sarazan et al., 2012). Quercetin suspension did not improve dP/dt max or cardiac contractility index, while nano-formulation containing the same dose of quercetin succeeded in restoring normal cardiac contractility. We cannot ignore the contribution of the plain nano-formulation in the previous effect as its administration alone without quercetin significantly increased cardiac contractility to some extent.

To calculate the diastolic function, the left ventricular diastolic time constant (*Tau*) was measured which was significantly prolonged in MetS animals. The nano-pharmaceutical formula of quercetin normalized *Tau* and its effect is similar to that of quercetin suspension or the plain nano-pharmaceutical formulation.

Notably, each MetS component such as dyslipidemia, elevated blood pressure, insulin resistance, and abdominal obesity, is recognized to damage the cardiac structure induce arrhythmias. Therefore, the coexistence of MetS and ECG deviation can possibly mean more risk of death due to sudden cardiac arrest (Abiodun et al., 2019). ECG of MetS animals revealed a significant prolongation of QTc, JT, and T peak-Tend intervals compared to control. On the other hand, the nano-formulation of quercetin augmented the effect of quercetin on ECG vectors reflecting ventricular activity which cannot be achieved by quercetin suspension alone.

Besides studying the effect of nano-formulation of quercetin on the electrical contraction of the heart, we also studied its effect on arterial waveform signal which reflects mechanical contraction of the heart. In the present work, MetS was associated with increased systolic and diastolic blood pressure but normal heart rate which is consistent with our previous study (Inanır et al., 2020). Oral administration of nano-formulation of quercetin reduced both systolic and diastolic blood pressure without affecting heart rate. Both quercetin suspension and the plain nano-pharmaceutical formulation reduced the elevated systolic and diastolic blood pressure but the effect of nano-formulation of quercetin on blood pressure is more prominent than their effects.

Moreover, three more parameters of arterial hemodynamics were evaluated in our present study; 1) pulse pressure, 2) the dicrotic notch pressure, and 3) the SDP difference. MetS was associated with increased pulse pressure, dicrotic notch pressure, and SDP difference, which is in accordance with our previous work (Azhar and El-Bassossy, 2015). The impairment in arterial hemodynamics observed in the present study increases the risk for cardiovascular diseases due to increased arterial stiffness and impaired cardiovascular performance and efficiency. The plain nano-pharmaceutical formulation or the nano-pharmaceutical formula of quercetin restored normal arterial hemodynamic measurements to that of control. While quercetin suspension improved pulse pressure and SDP difference but lacked a significant effect on dicrotic notch pressure.

Further, it is known that adipocytes produce signaling molecules, which are called adipokines or adipocytokines and influence insulin action, including adiponectin and TNF-α (Pittas et al., 2004). In the current study, the serum adiponectin level in MetS animals was noted to be considerably lower than that in the control group. Low plasma adiponectin levels are also usually found in many states that are generally linked to insulin resistance, such as cardiovascular disease (Kumada et al., 2003) and hypertension (Adamczak et al., 2003). Moreover, in the current study, MetS animals were distinguishable by their low-grade inflammation, as seen in the considerably higher TNF-α serum level. Notably, elevated levels were earlier noted in the inflammatory cytokine TNF-α in MetS animal models which were highly correlated with vascular complications (El-Bassossy et al., 2011; Mahmoud et al., 2012; Mahmoud et al., 2013).

The rise in cardiac TNFα caused by MetS was similarly eliminated by the three regimens but the reduction in adiponectin caused by MetS was eliminated only by quercetin suspension. The anti-inflammatory effect of quercetin in metabolic syndrome was previously demonstrated through its effect on TNF-α and adiponectin (Rivera et al., 2008; Wein et al., 2010; Zhang et al., 2018) and the anti-inflammatory effect of the PSO was also demonstrated in previous studies (Bardaa et al., 2016; Bardaa et al., 2020).

Finally, oxidative stress was also noted to contribute significantly in endothelial dysfunction and coronary artery disease (Heitzer et al., 2001). In the present study, the MDA level in MetS animals increased significantly compared to control animals which is in accordance with our previous study (Hassan et al., 2018). The nano-pharmaceutical formula of quercetin antioxidant activity was almost near that of the plain nano-pharmaceutical formulation and quercetin suspension. This confirms the anti-oxidant activity of the plain nano-pharmaceutical formulation which can be due to the presence of D-α-tocopheryl polyethylene glycol succinate (Engin, 2009). These results proved that the components of the plain nano-pharmaceutical formulation besides possessing antioxidant and anti-inflammatory activity, keep and maintain the antioxidant and the anti-inflammatory properties of quercetin itself and suggest its possible role in combating cardiovascular diseases.

The effect of the nano-pharmaceutical formula of quercetin on circulating insulin level and lipid profile, two of the main components of MetS, was also investigated in our present work. Our findings showed that the MetS was characterized by hyperinsulinemia and hypertriglyceridemia but normal cholesterol level. Hyperinsulinemia was eliminated similarly by the three regimens, but hypertriglyceridemia was reduced only by the plain nano-pharmaceutical formulation. These observations strongly suggest that the cardiovascular-protective effect exerted by SNEDDs of quercetin in part related to the normalization of circulating insulin level but still the main role in its cardiovascular protection is due to enhancing the bioavailability of quercetin-loaded SNEDDs preparation obvious from its prominent effect on different cardiovascular physiological indices previously discussed in the manuscript.

This is further explained by the left ventricle histological examination. MetS showed cardiac muscle fiber degeneration, myocardial necrosis, and inflammation which partially improved after the administration of quercetin suspension or the plain nano-pharmaceutical formulation. Noteworthy that the improvement in cardiac architecture of animals received plain nano-pharmaceutical formulation is more than that of quercetin suspension due to the reduction in the inflammatory cells infiltration and absence of necrotic tissue. The more improvement in heart tissue seen with the plain nano-pharmaceutical formulation is related to the antioxidant and anti-inflammatory components of the formulation which due to their combination in SNEDDs exhibited better bioavailability than quercetin suspension itself. However, the nano-pharmaceutical formula of quercetin succeeded in restoring the heart tissue pattern nearly similar to that of the control group, which proved the synergistic effect produced by both enhancing the bioavailability of quercetin and the beneficial effect of the SNEDDs components.

To the best of our knowledge, no report has utilized the combination of PSO (oil), TPGS (emulsifier), and PEG 200 (co-surfactant) as a promising carrier system for the improved delivery and synergistic efficacy of quercetin for protection from cardiovascular complications associated with MetS. The nano-pharmaceutical formula of quercetin consists of safe ingredients forming a nano-emulsion. PSO utilized is a natural oil and TPGS and PEG are approved by FDA. All ingredients of the formulation have a reported antioxidant activity. TPGS, the water-soluble derivative of natural vitamin E, has an amphiphilic excellent solubilizer, emulsifier, permeation, and bioavailability enhancer of hydrophobic drugs (Zhang et al., 2012; Guo et al., 2013; Yang et al., 2018). TPGS has a P-glycoprotein (P-gp) inhibition activity that could augment, with solubilizing ability, the efficacy of quercetin (Dintaman and Silverman, 1999).

Our recent study on the pharmacokinetic properties of quercetin-loaded SNEDDs preparation, showed 149.8% improvement in bioavailability of quercetin in SNEDDS relative to its suspension (Ahmed et al., 2021). The improvement in pharmacokinetic results of the quercetin-loaded SNEDDs formula could be a result of SNEDDS ability to enhance the permeability of the gut membrane for the transport of oily compounds (Cho et al., 2014). Furthermore, the instant self-emulsification offers quercetin in small globule solubilized form that massively increases the surface area for quercetin absorption in the gastrointestinal tract (Bu et al., 2014).

Taking into consideration that the enhanced effect of quercetin due to the current nano-formulation is not only due to enhanced bioavailability due to SNEDDs formation as the plain nano-formulation (with no quercetin) showed a marked cardioprotective effect. This points to a direct effect of the well-selected SNEDDS ingredients, Pumpkin seed oil, D-α-tocopheryl polyethylene glycol succinate (TPGS), and PEG 200, in cardiovascular protection. Pumpkin oil was chosen over other oils in the current SNEDDS preparation because of its anti-inflammatory effect (Fahim et al., 1995) while D-α-tocopheryl polyethylene glycol succinate was chosen based on its antioxidant activities (Engin, 2009). Several reports from our laboratories and others showed key roles of inflammation and oxidative stress in cardiovascular function associated with metabolic syndrome (El-Bassossy et al., 2009; El-Bassossy and Watson, 2015; El-Bassossy et al., 2018b).

In conclusion, the quercetin loaded SNEDDS is obviously more advantageous than the standard preparation of the drug in alleviating left ventricular and electro-mechanical cardiac dysfunction associated with MetS and in improving histopathological manifestations of cardiac damage incited by MetS. This beneficial effect is mediated through improving quercetin bioavailability supported by the well-selected SNEDDS ingredients, Pumpkin seed oil, D-α-tocopheryl polyethylene glycol succinate (TPGS) and PEG 200 which themselves showed marked cardioprotective effect.

## Data Availability

The raw data supporting the conclusion of this article will be made available by the authors, without undue reservation.
